# Parental influence on brown trout offspring immune cell composition: An infection study with *Tetracapsuloides bryosalmonae*

**DOI:** 10.1371/journal.pone.0308779

**Published:** 2025-09-24

**Authors:** Helena Saura Martinez, Gary Delalay, Stephanie Talker, Heike Schmidt-Posthaus

**Affiliations:** 1 Institute for Fish and Wildlife Health, Department of Infectious Diseases and Pathobiology, Vetsuisse Faculty, University of Bern, Bern, Switzerland; 2 Institute of Virology and Immunology, Bern, Switzerland; 3 Department of Infectious Diseases and Pathobiology, Vetsuisse Faculty, University of Bern, Bern, Switzerland; Benha University, EGYPT

## Abstract

Transgenerational immune priming (TGIP) is a phenomenon by which an initial exposure to a pathogen, here *Tetracapsuloides bryosalmonae* (causative agent of Proliferative Kidney Disease), stimulates the subsequent immune response to the same or a different pathogen in future generations. The impact of rearing conditions in previous generations, regardless of their exposure to the pathogen, on the immune cell composition and immune response in subsequent generations has not yet been investigated in the brown trout-*T. bryosalmonae* system. In the present work, we performed flow cytometry to analyze immune cell populations of brown trout. Parental generations (F0) differed in rearing conditions and exposure to the parasite. The study evaluated the baseline frequencies of IgM^+^ B cells, myeloid cells, and CD8^+^ T cells in the offspring (F1) young-of-the-year brown trout. Afterwards, F1 fish were experimentally infected with *T. bryosalmonae* spores and monitored during eight weeks post-infection. The kidney was identified as an immune-cell niche dominated by myeloid cells, which represent approximately two-thirds of the total immune cell population, along with a substantial proportion of IgM^+^ B cells. CD8^+^ T cells constitute only a minor fraction within this niche. As measured by flow cytometry, the immune-cell frequencies of offspring were largely unaffected by the parental rearing background (F0) and infection history. Parental history had no influence on the outcome of experimental infection. In *T. bryosalmonae* exposed animals, parasite concentration increased significantly over time. Moreover, a proportional increase in IgM^+^ B cells and a proportional decrease in myeloid cells over time was observed. However, the increase in IgM^+^ B cells was also detected in control animals. In conclusion, this study presents the first analysis of immune cell composition in F1 brown trout derived from parents reared under three distinct environmental conditions with varying parasite exposures. Our flow cytometry results highlight the need for alternative approaches to investigate transgenerational immune priming (TGIP) in brown trout.

## Introduction

The immune system exhibits considerable variability among individuals within the same species. Research on immune system variability in adults has underscored the significant influence of environmental factors on immune responses. Investigations into the epigenetic mechanisms governing immune responses have demonstrated that inter-individual variability in immune cell modifications predominantly arises from non-heritable factors [1]. As such, early-life influences can impact many of an organisms’ body functions, development, and life performance, including the immune system [2,3].

A hypothesis proposes that exposure of the parents to specific environmental stimuli earlier in life or during critical prenatal developmental periods exerts considerable influence on both short- and long-term health outcomes in offspring, by transfer of antibodies and through epigenetic modifications [2,4]. In oviparous animals, such as most bony fish species there is no direct antibody transfer from mother to progeny, although IgM immunoglobulins have been detected in unfertilized and unhatched rainbow trout eggs [5]. The early life represents a period of unique immune development during which the base for lifelong immunity is laid [6], and during which the conditions fish encounter can leave lasting effects in their physical, behavioral, and physiological traits [7].

A factor to consider in the context of immunity is parental effects. A parental effect refers to any influence exerted by parents on the phenotype of their offspring that cannot be attributed to the offspring’s genotype or to non-parental environmental factors [4]. Environmental conditions experienced by parents play a considerable role in disease and disease resistance in offspring, a phenomenon referred to as “transgenerational immune priming” (TGIP) [5]. TGIP is not mediated by the passive transfer of antibodies but depends on gene regulatory molecules deposited in germ cells (non-genetic or epigenetic information) [6], and may be even more crucial determinant of immunity in oviparous species, like fish or insects [7].

In our study, we selected the combination of brown trout and *Tetracapsuloides bryosalmonae*, provoking Proliferative Kidney Disease (PKD) [8,9], as the infection model to study TGIP effects. Brown trout are under pressure from global change dynamics [10] with wild stock of brown trout and other salmonid populations declining in Switzerland and worldwide [11–14]. This development is substantially attributed to PKD [13,15–17]. Although the environment inside the egg prior to hatching or before hatching is sterile, larvae or freshly hatched fish embryos experience a multitude of antigenic encounters; this happens especially in wild environments, where there is no option to evade the environmental pathogens, like the parasite causing PKD. However, there are scenarios where this situation is slightly different: in fish hatcheries, newly hatched fish are raised in pathogen-free facilities and usually do not face pathogens in those controlled environments.

PKD primarily affects the kidney, which in fish is the main hematopoietic organ. Brown trout have shown an adaptive immune response against re-exposure with the parasite [18–20] and survivors restore kidney structure and develop protective immunity against re-exposure [18,21,22]. Based on these assumptions, we postulate that brown trout previously exposed to the pathogen, such as wild trout from PKD-positive rivers, possess distinct immunological mechanisms compared to trout that have never encountered the pathogen, like farm-reared brown trout, thereby developing immune priming. We hypothesize that this immune priming can be transmitted across generations (via TGIP), as has been documented in other mammalian and insect species [5–7,23].

Fundamental questions were addressed in this study: Does parental (F0) rearing history have an influence on the immune cell composition of the offspring generation? Can parental exposure to the parasite have effects on the immune response of F1 (offspring) individuals to a parasitic infection?

To answer these questions, we selected three groups of brown trout with different backgrounds: wild brown trout (W), farmed brown trout raised at least two generations in the farm (F) and a wild:farm group whose wild parents were spawned and the fertilized ova raised in farm conditions (W:F). We explored the immune phenotype from these different backgrounds (W, F and W:F), and compared their immune responses towards an infection with *T. bryosalmonae* analyzing the frequency of IgM^+^ B cells, myeloid cells and CD8^+^ T cells.

IgM represents the major immunoglobulin present in teleost fish systemic immunity [24], and proliferation of IgM^+^ B cells has been observed in response to infections [25–27]. Myeloid cells have the ability to respond to infectious and damage-associated stimuli, by signaling through pattern-recognition receptors [28]. In rainbow trout, the innate immune response to PKD has been studied by flow cytometry (FCM) and immune gene PCR, showing predominantly a suppression of factors and immune genes involved in the innate immune system [29–31]. CD8 + cells have been less extensively studied and account for a very low proportion of cells (< 1.5%) in the anterior kidney of rainbow trout, as initially reported when the antibody was characterized by Takizawa et al. [32]. These cells represent a subset of T cells and are integral to the adaptive immune system.

Our experimental setup may offer insights into potential effects of TGIP on immune cell composition in the F1 generation. If parental exposure to the parasite influences offspring immunity, we might expect similar responses in groups W and W:F, given their shared parental origin. In contrast, group F, descended from farm-reared parents, could display distinct immune traits. Additionally, early-life environmental experiences might play a role: group W, having developed in a wild setting, may show immune characteristics that differ from those of W:F and F, which spent their early months in the controlled conditions of a farm environment. These contrasts could help tease apart the relative contributions of inherited versus early environmental influences.

Therefore, the objectives of this study were to investigate [1] potential differences in immune-cell frequencies among distinct groups of F1 brown trout with different parental rearing conditions (either wild or farm-originated parental generations) and different parental expositions to *T. bryosalmonae* (either exposed or non-exposed parental generation), and [2] potential differences in immune reaction in F1 brown trout infected with a subclinical dose of *T. bryosalmonae*.

By elucidating differences in immune-cell composition among these diverse rearing backgrounds and parental exposures, we aim to contribute to a better understanding of the possible effects of the rearing environment on the development of the immune system and of immune reactions in brown trout, a fish species of ecological and economical importance.

## Materials and methods

### Experimental setup and conditions

The experiment was performed under controlled laboratory conditions. Brown trout from different rearing histories and environments were used: Wild group (W), Wild:Farm group (W:F) and Farm group (F):

- Wild group (W): this group consisted of young-of-the-year (YOY) brown trout (F1 generation) that were electrofished during spring 2021 in known PKD-positive Swiss rivers over a stretch of 100m each (*Brübach*, between 47.471195 °N/ 9.136465 °E and 47.473303 °N/ 9.140526 °E; and *Rörlibadbach,* between 47.473019 °N/ 9.136563 °E and 47.478731 °N/ 9.134755 °E). The parents (F0 generation) were born and raised in the wild. These waterbodies are monitored and known to be positive for PKD for generations, as year after year, 100% of investigated trout are tested positive for *T. bryosalmonae* (H. Schmidt-Posthaus, unpublished data). Therefore, we assume that the F0 generation has already faced the infection with *T. bryosalmonae* in their early life.

- Wild:Farm group (W:F): the parents (F0 generation), wild female and male adult brown trout, were fished in the same PKD-positive river system (*Brübach*, coordinates between 47.462306 °N/ 9.122916 °E and 47.481201 °N/ 9.159397 °E) and spawned in autumn 2020. Fertilized eggs were transferred to a farm facility where they were raised until spring 2021 (F1 generation born and raised in farm). As for the W group, we assume that the F0 generation has also been exposed or faced an infection with the parasite earlier in life due to their origin from the same waterbody.

- Farm group (F): this group consisted of fish whom ancestors over two generations (F0 and the previous generation) were born and raised in a Swiss fish farm, with both the F0 individuals and their ancestors never having been exposed to the parasite before. The genetic line originated from the same river system (*Brübach*). Ova and sperm of adults were spawned in autumn 2020 and the fertilized eggs transferred to the same facility as the W:F group where they were raised until spring 2021 (F1 generation born and raised in farm).

**Fig 1:**
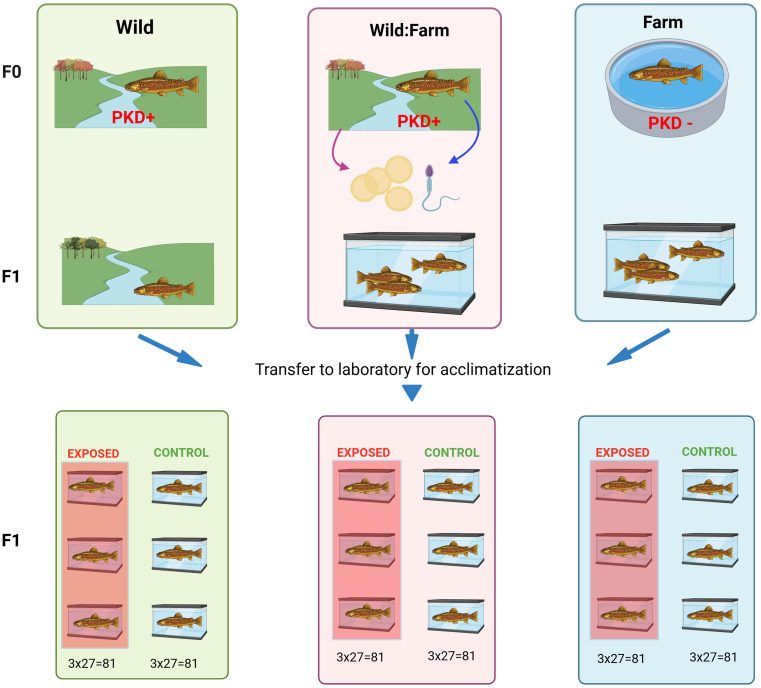
Schematic setup of infection trial with three brown trout populations.

All YOY (250 animals per group) were transferred in May 2021, when fish were approximately 3 months old, to the facility of the Institute for Fish and Wildlife Health (FIWI), University of Bern and held in 21 independent, separate 38L tanks, with separated water supply. The fish were acclimatized to laboratory conditions for four months prior to experimental procedure. Clinical signs and mortalities were assessed daily. After acclimatization, each group of fish (W, W:F, F) was distributed into six 38 L aquaria, with equal fish numbers (n = 29 per aquarium); three aquaria for biological control replicates and three aquaria for exposed replicates (Fig 1). At day 0, two fish per aquarium were subjected to a health check, resulting in 27 fish per aquarium at initiation of experiment.

Fish were maintained at constant 16°C with a separated flow-through water system (flow rate 40 L/min, no contact between aquaria) per aquarium and 12:12h light:dark photoperiod. This temperature is above the known 15°C threshold for PKD outbreaks and the lack of temperature-variability during the study should minimize the temperature stress for the animals. The fish were fed 2.5% of their body weight twice a day with a commercial diet and were additionally given live artemia in the first month after their arrival. All procedures complied with the Swiss legislation for animal experimentation guidelines, and they were previously approved by the cantonal veterinary office (Bern, Switzerland) (Authorization number BE25/2021).

The F0 generation represents the parental generation, while the F1 generation represents their offspring. The F0 individuals from the Wild (W) and Wild:Farm (W:F) groups originated from rivers where *T. bryosalmonae* is known to be present (PKD+). Conversely, the F0 individuals from the Farm (F) group are derived from a farm, with both the F0 individuals and their ancestors never having been exposed to the parasite (PKD-). For the W:F group, F0 individuals were spawned, their ova fertilized, and the resulting F1 eggs raised on a farm. The F1 generation of the W group was captured in the wild and then directly transferred to FIWI for acclimatization. F1 individuals from the W:F and F groups were raised on the same farm and subsequently transferred to FIWI at the same time point as the F1 individuals from the W group were captured. Upon arrival at the FIWI, all fish were evenly distributed into 38 L aquaria, with three replicates for each origin (W, W:F, F) and treatment (exposed or control). For the experiment, 27 fish were present per aquarium. Created with Biorender.com.

### Fish health check and necropsy

At arrival, two fish per aquarium were randomly selected for an initial health check and euthanized with an overdose of tricaine (150 mg/L tricaine methanesulfonate; Tricaine PHARMAQ®, Pharmaq). A complete necropsy was performed and macroscopical changes on external and internal organs were documented. An external (skin and gills) and internal (gut) analysis for detection of parasites in a native smear, and samples from spleen, liver and kidney were cultured on blood agar plates and bromothymol blue–lactose–agar plates (Bio Mérieux, Switzerland) and incubated at 22°C for 48 hours for bacteriological analysis. Due to the prominent role flavobacteria play in Swiss aquaculture and in the wild, samples of gill and spleen were cultivated on specific agar plates [33] for 5 days at 15°C. The kidney was dissected and placed in RNAlater (Qiagen, Basel, Switzerland) for a consecutive qPCR analysis for the presence of *T. bryosalmonae.* Animals found dead during the experimental phase were subjected to a full necropsy and to bacterial and parasitological examinations as described above.

### Parasite exposure

Freshwater bryozoans were collected from Swiss rivers known to be endemic for the parasite (*Alte Aare,* coordinates: 590781/ 217845 and *Furtbach*, coordinates: 2670258/ 1255599), transferred to the FIWI and screened for infective parasite sacs under a stereomicroscope. To release *T. bryosalmonae* spores, bryozoan zooid tissue was disrupted by grinding, and the homogenate kept in original river water at room temperature for 1 h until addition to the aquaria. From two 2 mL homogenate, DNA was extracted and qPCR [34] was performed as previously described [34] to investigate presence of *T. bryosalmonae* DNA and to calculate infection dose. The parasitic homogenate had a concentration of 1 000–10 000 parasite DNA copies/ µL. Prior to exposure, the water flow was stopped, aeration increased, and tank water lowered to 20% (approx. 7.6 L). The parasite homogenate was diluted to obtain equal volumes of 24 mL of the homogenate containing 2x10^4^ copies of parasite DNA each to distribute to all exposed replicates. After 1.5 h, the water flow was restarted. Procedure was performed simultaneously for controls without addition of parasites. This procedure was repeated on three consecutive days with the same doses.

### Fish sampling

Prior to exposure to the parasite, one fish per tank was euthanized and analyzed for the presence of *T. bryosalmonae* DNA. The infection experiment lasted for 54 days, and 7 samplings were performed at days 4, 11, 19, 25, 35, 41, and 54 post exposure (dpe).

Fish sampling procedures were carried out in all control and exposed groups simultaneously. Two fish per aquarium (6 fish per control and 6 fish per exposed group) were sampled: one fish for immune cell analysis (flow cytometry, FCM) and one fish for molecular investigations (RT-qPCR), respectively, at each time point. Due to the small size of the fish at the start of the study, the whole fish were used for either the cellular or the molecular methods. We had a total of three biological replicates x three groups of origin (W, W:F, F) x two treatments (control/ exposed) for each method. At each sampling time point, fish were euthanized by immersion in an overdose of tricaine (150 mg/L tricaine methanesulfonate; Tricaine PHARMAQ®, Pharmaq). Following euthanasia, the entire kidney was removed. Kidney tissue used for RT-qPCR was placed in RNAlater (Qiagen, Basel, Switzerland) and stored at −20°C until further analysis. Kidney tissue used for FCM was processed immediately and cells were prepared as described below. At 25 dpe, a sample was used for sc-RNA-sequencing (not described in this manuscript) instead of flow cytometry.

### Isolation of kidney leukocytes for flow cytometry

Kidney leukocytes were isolated as described earlier [31,35]. Briefly, the complete kidney was removed and passed through a mesh filter with a pore size of 105 μm with Leibovitz’s (L-15) medium (Thermofisher, Reinach, Switzerland) containing 10% fetal calf serum (FCS). Cell suspensions were layered onto a sterile, isotonic Ficoll gradient (Ficoll-paque Plus, GE Healthcare Bio-Sciences AB, Sweden) with a density of 1.077 g/mL and spun at 750 x g for 40 min at 4°C to separate leukocytes from erythrocytes. Leukocytes at the Ficoll/medium interphase were aspirated, washed in L-15 medium and centrifuged at 290 x g at 4°C for 10 min. Cells were counted manually and in an automated cell counter (DeNovix® CellDrop Automated Cell Counter, NC, USA). A total amount of between 1 and 2 x 10^6^ total cells per animal was obtained.

### Flow cytometry

Total kidney leukocytes were split among three stainings. We used a U-bottomed 96-well plate for the staining with three monoclonal antibodies in a total volume of 20 μL/well: For detection of B cells, two monoclonal antibodies (MAbs) Mab1.14 (recognizing IgM) and MAbN2 (recognizing the κ -like Ig light chain, kLC) were combined in one staining as previously described [36,37]. Due to high variability of the ĸlight chain staining, only IgM + B cells were included in the final analysis. Myeloid cells were stained with the Mab myeloid-21 [38,39]. Additionally, a Mab staining rainbow trout CD8^+^ T cells was used as described before [32].

Cells were incubated with primary antibodies (Ab) for 20 min at 4°C (Table 1). After this, cells were washed twice (4 min at 400 x g, 4°C) with PBS and incubated for 20 min at 4°C in the dark with the corresponding secondary antibodies. After incubation with secondary Abs, cells were washed again twice with PBS and kept on ice until flow cytometric analysis. A total of 1 x 10^5^ cells was acquired per sample on a BD SORP LSR II flow cytometer (San Jose, CA, USA) equipped with three lasers (488 nm, 633 nm and 407 nm). Data were analyzed by FlowJo™ v10.8 Software (BD Life Sciences).

**Table 1:** Antibody reagent list.

Antibody	Specificity	Stained cells	Reference	Clone	2ary Ab	Fluorochrome	Provider 2ary Ab
Mouse anti-rainbow trout MAb-1.14	Trout membrane-bound IgM	IgM^ +^ B cells	[40]	1.14	goat anti-mouse IgG1	AF647	Molecular Probes
Mouse anti-rainbow trout MAbN2	Ig ĸ-light chain	Ig ĸ-light chain^+^ cells	[36]	5G1	goat anti-mouse IgG2b	AF488	Molecular Probes
Rat anti-rainbow trout CD8	CD8	CD8α^ +^ T cells	[32]	7α 8c	donkey anti-rat IgG-RPE-conjugated^1^	PE	Jackson ImmunoResearch Laboratories
Mouse anti- rainbow trout myeloid-21^2^	myeloid cells	Myeloid cells	[38,39]	ASK H	goat anti-mouse IgG2b	AF488	Molecular Probes
Live-dead discriminator (Near-IR)		Dead cells					

^1^RPE = R-Phycoerythrin conjugated fragments; ^2^myeloid cells: larger granular cells of the myeloid lineage (large granulocytes, monocytes/macrophages) [31,41].

**Fig 2:**
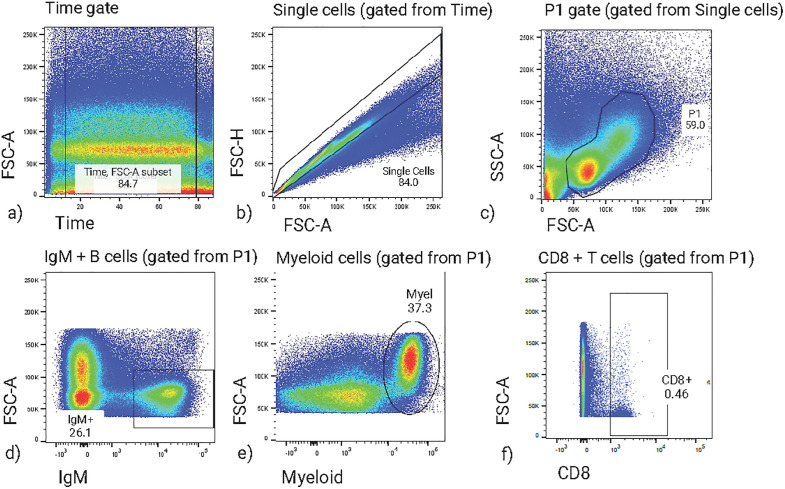
Flow cytometry gating strategy for analyzing IgM^+^, myeloid, and CD8^+^ subsets of kidney from brown trout. Immune cells from brown trout freshly isolated from kidney were stained for flow cytometry. (a) A “time” gate was used to gate out irregularities in sample acquisition. (b) Doublets were excluded based on the FSC-A vs. FSC-H plot. (c) Based on FSC-A vs. SSC-A, events of interest (including lymphocytes and myeloid cells) were selected. (d) IgM expression was plotted against FSC-A to target the B cell population (IgM^+^ FSC-A^low^). (e) Myeloid cells were gated as myeloid-marker^+^. (f) CD8 expression was plotted against FSC-A to target CD8 T cells. Frequencies within parent populations are indicated inside the plots.

The primary Abs used in this experiment were originally designed specifically for rainbow trout. We tested their specificity towards brown trout leukocytes in a previous pilot experiment and performed the titrations of the individual Abs to determine the best concentration. For every secondary Ab, unspecific staining was assessed by a conjugate control (trout kidney leukocytes incubated with secondary Ab only). The gating strategy is shown in Fig 2.

The specificity of monoclonal antibodies together with selected fluorochromes used in the current study is listed. All the antibodies used were directed against known surface markers of rainbow trout.

### Kidney leukocyte gating strategy

We analyzed immune cells of the B-cell lineage with the anti-IgM antibody and of the myeloid cell lineage with an anti-myeloid antibody. Positively stained IgM^+^ and myeloid^+^ cells were detected with Alexa Fluor-647-conjugated goat anti-mouse IgG1 (Molecular Probes) and Alexa Fluor 488-conjugated goat anti-mouse IgG2b (Molecular Probes), respectively. Positively stained CD8^+^ T cells were detected with RPE-conjugated anti-rat IgG (Jackson ImmunoResearch Laboratories). The gating strategy used to evaluate MAbs is shown in Fig 2. Further details of conjugate controls and full stainings are available in Supplementary Material (S1 Fig). A common large gate was used to determine the frequencies of immune-cell populations (CD8 + , IgM + , myeloid+) that excluded FSC-A-low cells (presumably dead cells). Percentages of populations within the lymphocyte gate and backgatings of marker positive populations on the FSC-A/SSC-A plot are shown in the Supplementary Material (S2 Fig) for three exemplary samples from day 4 post infection.

### DNA extraction and qPCR for determination of parasite kinetics/ prevalence

DNA was extracted from whole kidney of control and exposed animals using DNeasy blood and tissue kit (Qiagen, Basel, Switzerland) following the manufacturer’s guidelines. DNA concentration and quality were checked with NanoDrop™ One UV-Vis Spectrophotometer (Thermo Fisher Scientific). qPCR was performed targeting the *T. bryosalmonae* 18S rRNA gene (Acc. N.: AF190669) using the specific TaqMan method as reported by Bettge et al. [34]. Briefly, each reaction was carried out in a final volume of 20 µl containing 1X TaqMan universal Master Mix (Applied Biosystems), 0.3 μM of each primer (PKDtaqf1: 5′–GCGAGATTTGTTGCATTTAAAAAG–3′ and PKDtaqr1: 5′– GCACATGCAGTGTCCAA TCG–3′), 0.2 μM of the probe PKD (5′– CAAAATTGTGGAACCGTCCGACTACGA–3′), 1× of internal control Exo IPC Mix, 1× of IC DNA (TaqMan Univ. MMix w Exog IntPostC, Applied Biosystems), and 2 μl of template DNA. The qPCR was performed using a MicPCR machine (Bio Molecular Systems, Australia). To estimate the DNA concentration, positive samples were re-tested using a standard curve. A standard curve was generated for each run using five dilutions from 10^−3^ down to 10^−9^ ng/µL of synthesized g-blocks. The regression coefficient of the standard curve had to be between −3.6 and −3.0, and the coefficient of variation between triplicates below 25% [42]. If these criteria were not met, the sample was tested again. Non-target controls (nuclease free water) within the qPCR never showed any amplification.

We used a sample from fish positive for *T. bryosalmonae* DNA from internal experiments as a positive control that always showed amplification.

### Data analysis and visualization

The data were imported in R 4.5 [43] with packages readxl 1.4.3 [44]. Data manipulation was performed with the packages tidyr 1.3.1 [45] and dplyr 1.1.4 [46]. Analyses were performed with the base R packages. Confidence intervals for proportions were calculated using the Clopper–Pearson method and computed in R with the package PropCIs 0.3−0 [47]. Linear regressions were computed with the base R packages and the package parsnip 1.3.1 [48] and the results explored with the package broom 1.0.8 [49]. Figures were computed in R with the packages ggplot2 3.5.2 [50,51], ggbeeswarm 0.7.2 [52], ggtext 0.1.2 [53] and cowplot 1.1.3 [54].

## Results

### Mortality

In the acclimatization period, there was a significant difference in mortality between the group W and both other groups. In group W, 33 out of 250 fish died (13.2%, 95% CI: 9.3%–18.0%), while only 10 out of 250 fish in the group W:F (4.0%, 95% CI: 1.9%–7.2%, χ^2^ = 12.315, p ≤ .001) and 6 of 250 animals in the group F died (2.4%, 95% CI: 0.9%–5.2%, χ^2^ = 18.8, p ≤ .001). There was no significant difference between groups W:F and F (χ^2^ = 0.5811, p = .45).

Signs of disease were absent during the whole experimental period. During the experiment, four control fish (one fish W group, three fish W:F group) and one exposed fish (W:F group) died. The dead fish showed no signs of disease. Those fish were subjected to a full necropsy and bacterial and parasitological examinations were performed. These dead animals did not show pathological changes, and no bacteria or parasites were detected. qPCR for *T. bryosalmonae* was negative.

### Initial health check

We did an initial health check of 2 fish per aquarium at arrival. We detected an initial infection with *Gyrodactylus* sp. on the skin of fish from the W group. We treated the wild brown trout for three consecutive days with 3% salt during 20 min that we progressively increased to 1 hour along the three therapy days. No bacteria were detected in kidney, liver, or spleen. No animal showed amplification in the qPCR against *T. bryosalmonae*.

### Frequency of immune-cell populations

Using flow cytometry, we determined the frequency of main immune cell populations (IgM^+^ B cells, myeloid cells and CD8^+^ T cells) within head and posterior kidney leukocytes isolated from brown trout of the three rearing backgrounds (W, W:F, F).

**Fig 3:**
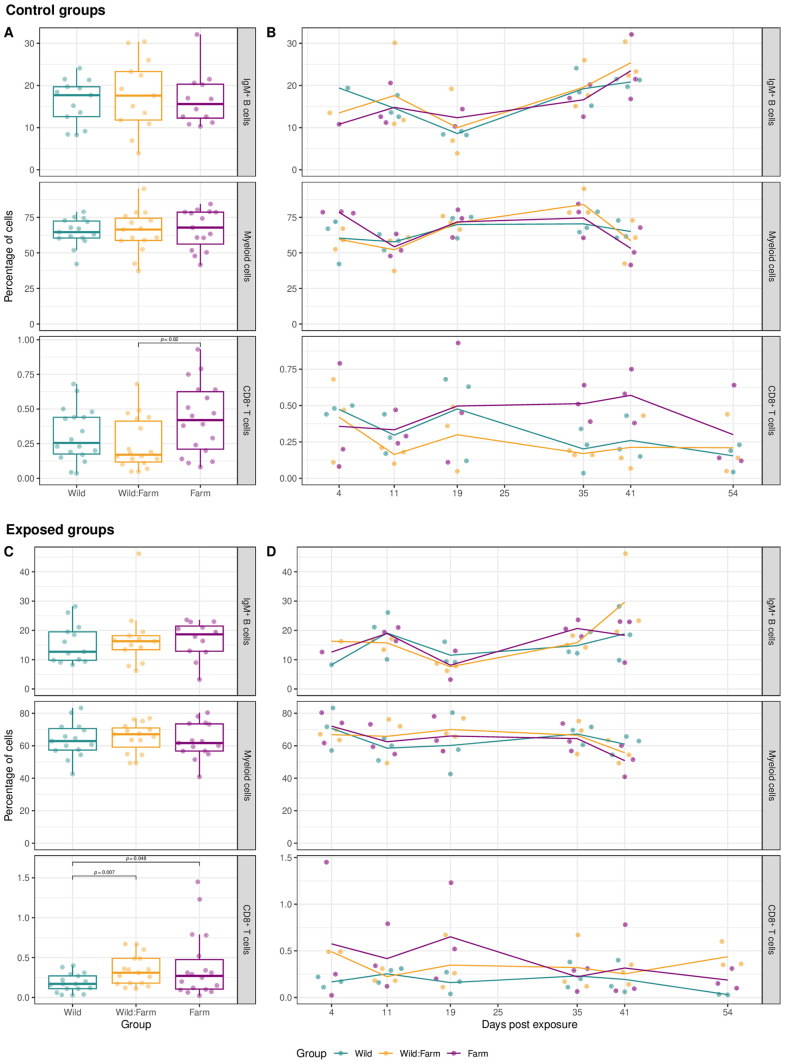
Frequencies of IgM^+^ B cells, myeloid cells and CD8^+^ T cells of control and exposed brown trout in Wild, Wild:Farm and Farm groups. **A)** Boxplots show the mean frequencies of IgM^+^ B cells, myeloid cells and CD8^+^ T cells gated from the P1 gate by flow cytometry (Fig 2) for the control group (A) and the exposed group **(C)**. Individual data points are represented as scattered dots. Results from all the control Wild, Wild:Farm and Farm groups from all sampling timepoints are shown. B + D) The line represents mean values of control animals **(B)** and exposed animals **(D)** (n = 3 fish per group and time point) over the course of the experiment. Individual data points are represented as scatter dots for each timepoint. Due to technical problems on sampling day 54, there are no data of IgM^+^ and myeloid cell populations.

For the control group, IgM^+^ B cells ranged from 3.9% to 32.1% (mean = 16.9%, sd = 6.7%), myeloid cells from 37.3% to 95.1% (mean = 65.4%, sd = 12.7%), and CD8^+^ T cells from 0.04% to 0.93% (mean = 0.33%, sd = 0.22%) (Fig 3A). We have not observed significant differences in the frequency of IgM + B cells (W: mean = 16.1%, sem = 1.48%, n = 13; W:F: mean = 17.8%, sem = 2.34%, n = 13; F: mean = 16.7%, sem = 1.81%, n = 12) and myeloid cells (W: mean = 64.7%, sem = 2.48%, n = 15; W:F: mean = 65.1%, sem = 3.81%, n = 15; F: mean = 66.5%, sem = 3.57%, n = 15) between the different rearing backgrounds W, W:F, and F (Table 2 and Fig 3A). Before Bonferroni correction, CD8 + T cells showed a difference between W:F and F groups (W: mean = 0.31%, sem = 0.05%, n = 18; W:F: mean = 0.25%, sem = 0.04%, n = 18; F: mean = 0.43%, sem = 0.06%, n = 18).

**Table 2:** Minimal estimated prevalence for each aquarium at the end of the study.

Group	Tank	Number of positive fish	Number of fish sampled	Minimal prevalence (%)	Confidence interval (Clopper–Pearson) (%)
W	Replicate 1	6	7	85.7	42.1–99.6
	Replicate 2	1	7	14.3	0.4–57.9
	Replicate 3	2	7	28.6	3.7–71.0
W:F	Replicate 1	3	7	42.9	9.9–81.6
	Replicate 2	5	7	71.4	29.0–96.3
	Replicate 3	4	7	57.1	18.4–90.1
F	Replicate 1	1	7	14.3	0.4–57.9
	Replicate 2	3	7	42.9	9.9–81.6
	Replicate 3	4	7	57.1	18.4–90.1

When the control group was analyzed in the time course (starting at 4 dpe), IgM^+^ B cells showed a statistically significant increasing trend (*F*([1],[36]) = 12.72, p = .001, adjusted R² = .24, coeff = 0.26%, SE = 0.07%), while no statistically significant trends were observed for myeloid cells (*F*([1],[43]) = 0.65, p = .426) and for CD8 + T cells (*F*([1],[52]) = 2.20, p = .144) (Fig 3B).

In the exposed group, IgM^+^ B cells ranged from 3.2% to 46.2% (mean = 16.5%, sd = 7.7%), myeloid cells from 40.9% to 83.3% (mean = 63.9%, sd = 10.1%), and CD8^+^ T cells from 0.02% to 1.45% (mean = 0.31%, sd = 0.28%) (Fig 3C).

When the exposed group was analyzed in the time course (starting at 4dpe), a statistically significant increasing trend (before Bonferroni correction) was found for IgM^+^ B cells over the course of the infection (*F*([1],[36]) = 4.89, p = .033, adjusted R² = .10, coeff = 0.20%, SE = 0.09%) and a statistically significant decreasing trend (before Bonferroni correction) for myeloid cells (*F*([1],[43]) = 4.66, p = .036, adjusted R² = .08, coeff = −0.22%, SE = 0.10%), while no statistically significant trend was observed for CD8^+^ T cells (*F*([1],[50]) = 1.74, p = .193) (Fig 3D).

In exposed animals, we observed a significant difference before Bonferroni correction in the proportion of CD8^+^ T cells between the groups W and W:F and the groups W and F (W: mean = 0.18%, sem = 0.03%, n = 17; W:F: mean = 0.34%, sem = 0.05%, n = 17; F: mean = 0.39%, sem = 0.10%, n = 19) (Table 2). We have not observed significant differences in the frequency of IgM^+^ B cells (W: mean = 15.5%, sem = 1.86%, n = 13; W:F: mean = 17.1%, sem = 2.77%, n = 13; F: mean = 16.9%, sem = 1.82%, n = 12) and myeloid cells (W: mean = 63.5%, sem = 2.78%, n = 15; W:F: mean = 65.0%, sem = 2.38%, n = 15; F: mean = 63.2%, sem = 2.81%, n = 15) between the different rearing backgrounds W, W:F, and F (Table 2 and Fig 3C).

There was a significant difference before Bonferroni correction in the CD8 + T cells populations throughout the experiment between control and exposed groups for the Wild group (p = .021, S1 Table, S4 Fig), but no other significant difference could be found.

### *T. bryosalmonae* concentration

There were no visible macroscopical changes typical for PKD, like kidney enlargement, in any of the exposed animals, at any time point. This, together with the absence of mortality, indicates a subclinical infection.

Prior to initiation of the experiment, no animal showed PCR amplification for *T. bryosalmonae*.

**Fig 4:**
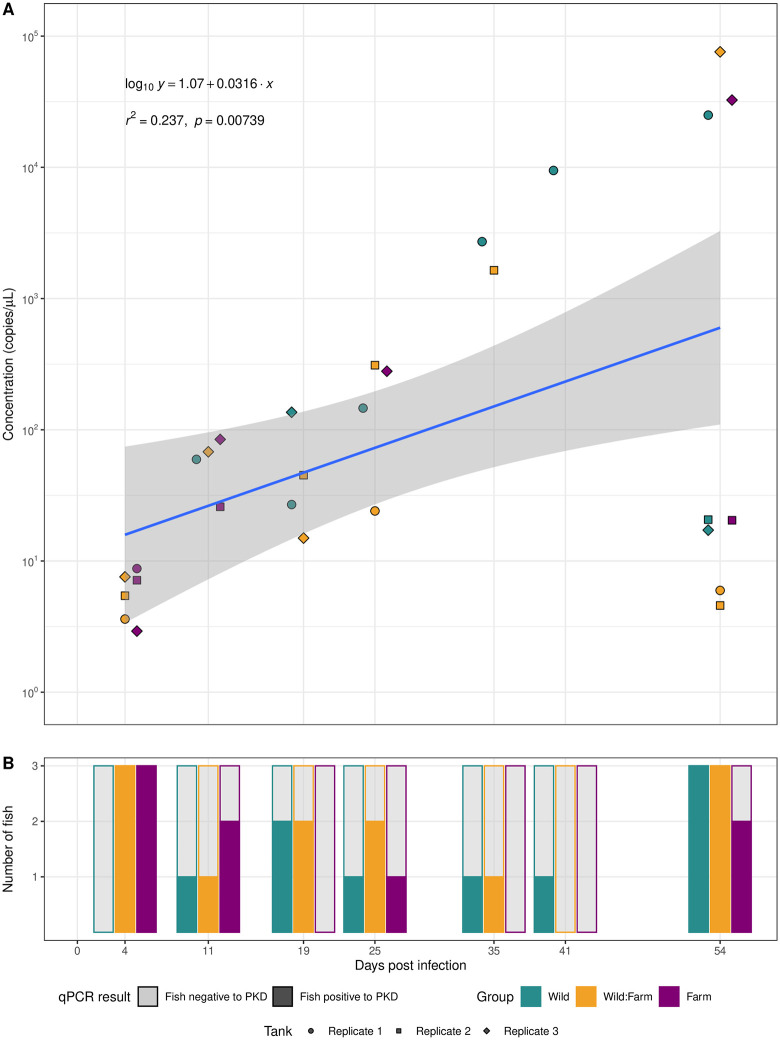
qPCR results of *T. bryosalmonae* DNA amplification on brown trout kidney and estimated concentration.

During the infection experiment, one brown trout per sampling timepoint and per aquarium was euthanized and its kidney analyzed for *T. bryosalmonae* DNA. qPCR results are shown in Fig 4. In total, 9 trout from W, 12 from W:F and 8 from F out of 21 sampled fish in each group were positive for *T. bryosalmonae* DNA. The differences were not significant (W & W:F: χ^2^ = .381, p = .537; W & F: χ^2^ < .001, p > .999; W:M & F: χ^2^ = .859, p = .354).

Concentration of parasite DNA in kidney tissue increased continuously over time and ranged from 1.58x10^-9^ to 4.10x10^-5^ copies/μL (Fig 4).

Represented are the results of *T. bryosalmonae* DNA amplification assessed by qPCR on brown trout kidney of exposed fish with the estimated concentrations of the positive samples. All sampling timepoints are represented (Y-axis). The different groups are represented with colors: the Wild group is green, the Wild:Farm group is yellow, and the Farm group is magenta. For each group (W, W:F, F), there were three replicates, represented with different point shapes. A) Estimated concentration in copy number/μL of *T. bryosalmonae* DNA in exposed brown trout. The different shapes represent the replicate tank from which the fish originated: circle is replicate 1, square is replicate 2, diamond is replicate 3. B) qPCR results of exposed brown trout sampled during the study. Three fish per group (one from each replicated tank) were sampled at each sampling timepoint. In total, *T. bryosalmonae* DNA was amplified in 29 out of 63 sampled exposed trout. Grey bars represent fish from which no *T. bryosalmonae* DNA could be amplified, while colored bars represent fish from which *T. bryosalmonae* DNA was successfully amplified.

### Infection prevalence

Considering the duration of disease, we expect that infected fish detected before the end of our study would have stayed infected until the end of our study, while negative fish could have potentially developed disease before the end of the study. Therefore, we can estimate the minimal prevalence reached for each aquarium at the end of our study (Table 2).

Point prevalence requires data originating from a single point in time, but our data was collected over the course of several weeks. Due to the disease duration, fish tested positive would still be considered positive at the end of the study, while fish tested negative could potentially develop disease before the end of the study. The numbers are therefore to be considered as minimal prevalence at the end of the study (54dpe), as the real prevalence could potentially be higher if some negative tested fish prior to 54dpe would have developed the disease.

## Discussion

Immune responses exhibit significant variability among individuals. Recent research over the past decades has expanded our understanding of the effects of non-genetic inheritance on an individual’s immune system [6,23,55] and has elucidated how rearing conditions influence an individual’s response to pathogens [5,56].

**In our study, we showed cross-reactivity of monoclonal antibodies developed for rainbow trout against IgM + B cells, myeloid cells and CD8 + T cells in brown trout.** In this study, we employed rainbow trout-specific antibodies in brown trout tissue for the first time. Antibodies targeting IgM^+^ B cells (clone 1.14) and myeloid cells (clone ASK H) produced reliable and robust results. The mAb against CD8 (clone 7α 8c) only stained a very small population of between 0.02 and 1.45% of the total immune cells of the kidney. The study which developed the CD8 antibody used here reported consistent low amounts of CD8^+^ cells in the anterior kidney of rainbow trout (exact numbers not reported) and also in peripheral blood (with frequencies of approximately 0.3%) [38]. According to these findings, the results obtained in our experiment with brown trout cells seem plausible. Other literature of rainbow trout PKD experiments in which this antibody was used did not report the frequency of CD8^+^ T cells [41], but evidenced upregulation of CD8 transcripts. The kLC staining showed specific reactivity, however due to inconsistencies in the gating strategy, in particular difficulties to define negative cells across samples (S3 Fig), the kLC staining was excluded from further analysis.

**On average, immune cells from brown trout kidney were composed of 17% IgM**+ **B cells, 65% myeloid cells, and 0.3% CD8**+ **T cells across rearing backgrounds.** This finding is consistent with other studies focused on rainbow trout that have shown MHCII gene expression (indicating macrophage presence) at much higher levels than expression of lymphocyte marker genes soon after hatching [57]. The authors who reported the establishment of the CD8 antibody used in this experiment [32] were surprised by the low levels of cells staining positive with this antibody in the anterior kidney and peripheral blood of rainbow trout, observing frequencies of approximately 0.3% within immune cells. This finding contrasts with mammals and other vertebrates, where the population of CD8 T cells is markedly higher in both lymphoid organs and peripheral blood, like mouse spleen with 10% of these cells [58] or human spleen with around 19% [59]. The percentage of some immune cell lines of hematopoietic organs is determined in other fish species [60], but to our knowledge, there are no studies showing the proportion of hematopoietic cell lines in the kidney of brown trout and no published information towards the amount of myeloid cells vs. lymphocytes in the kidney. In the present study we applied a cutoff based on forward scatter area (FSC-A) to gate out putative dead cells. Especially cells that stained positive with the CD8 antibody displayed a relatively low forward scatter, which may be indicative of dead cells. For a more accurate estimation of immune-cell frequencies in kidney of brown trout, a live-dead staining should be included for future analyses.

**Parental rearing conditions and early-life environment did not have a marked impact on the investigated frequencies of immune cells in control animals.** We investigated the frequency of IgM^+^ B and myeloid cells in the kidney up to 41 dpe and CD8^+^ T cells up to 54 dpe for the three brown trout groups from the W, W:F and F origin. There were no differences at cellular level regarding the frequency of IgM^+^ B or myeloid cell populations in the kidney (hematopoietic organ in fish). CD8^+^ T cells showed differences between W:F and F group, but only before Bonferroni correction. According to these results, the parental rearing conditions or the early-life environment (before transfer into the laboratory) of the studied fish do not seem to significantly alter the overall frequency of the investigated immune cells. Biological differences between the groups may be too subtle. This hypothesis is supported by the fact that – in contrast to the cellular level – differences between groups of different rearing backgrounds seem to be present on gene expression level (Ord et al., Coxon et al., unpublished data).

However, although the difference between groups was negligible, it is interesting to note that variation between individuals was relatively high, with a standard deviation of 6.7% (mean = 16.9) for the IgM^+^ B cells, 12.7% (mean = 65.4%) for myeloid cells and 0.22% (mean = 0.33%) for CD8^+^ T cells. These findings suggest that individual environmental factors may have a stronger influence on immune cell composition than parental origin. This effect appears particularly pronounced in the Wild group, which is exposed to more variable environmental conditions compared to farm-raised brown trout. However, this hypothesis requires further validation, for example through group-level comparisons at the genetic and molecular levels.

**A temporal increase in B cells and decrease in myeloid cells was seen in exposed brown trout.** In our infection experiment, we worked with a subclinical infection without PKD-related organ changes nor mortalities. In clinical PKD infections, immune responses are altered and unregulated, typically manifesting as severe and dysregulated reactions: studies have demonstrated that such disease manifestations result in disrupted B-cell responses and suppressions of the innate immune response [29,31,61], characterized by significant upregulation of anti-inflammatory cytokines. Bailey et al. [31,62] employed the same technique in rainbow trout and observed an increase in lymphocytes and a decrease in larger granular myeloid cells from the second week post-exposure (wpe). This is in accordance to our results in subclinically exposed brown trout. We encountered a significant proportional increase in IgM^+^ B cells and a significant decrease in myeloid cells between the third and seventh wpe. These results support the hypothesis that *T. bryosalmonae* infections lead to B cell activation and suppression of macrophage/monocyte lineage. The importance of B cells for protecting the brown trout host against *T. bryosalmonae* invasion was also shown by Shivam et al. [63]. However, the results were only significant before Bonferroni correction and should therefore be validated by further studies. Additionally, in our study, the temporal increase in IgM^+^ B cells was also detectable in control animals, indicating that the temporal B cell increase is rather due to B cell maturation. No temporal change in CD8^+^ T cells was obvious in our study. In contrast, in rainbow trout, an increase of CD8^+^ T cells was detected 10 wpe [35]. Most studies in brown trout have focused on immune gene transcripts rather than flow cytometric analysis of immune cells, with evidence of upregulated immune genes in brown trout during disease progression at 30 dpe [64], whereas rainbow trout show sooner upregulation at 14 dpe [31]. Compared to brown trout, rainbow trout exhibit distinct reaction patterns and timing in their immune gene responses and immune-cell frequencies when infected with *T. bryosalmonae*.

The absence of clinical signs in exposed brown trout in our study may be attributed to the fact that brown trout are native hosts of *T. bryosalmonae*, unlike rainbow trout [65]. Given the long-standing presence of *T. bryosalmonae* in Europe [17,66], co-evolution may have occurred, allowing brown trout to tolerate moderate infections under optimal conditions. In the absence of additional stressors, such as elevated temperatures [31], poor water quality [67], or limited food, such infections may not trigger an overwhelming immune response. None of the published studies on brown trout, including our own, reported macroscopic signs of disease, such as renomegaly. This contrasts with findings in rainbow trout, which exhibited renomegaly starting from 7 wpe [35]. However, kidney histopathology and parasite detection can be present even without macroscopical evidence and they have been shown to be influenced by the infection concentration of the parasite: a low-concentration exposure (5.4 x 10^6^ total copies) showed delayed parasite detection and reduced histopathology, whereas high-concentration exposure (42 x 10^6^ total copies) and repeated exposures exhibited increased immunopathology at their first sampling 30 dpe [68]. Our experiment was performed with between 20 and 200 x 10^6^ total copies during three consecutive days, thus allowing for pathological changes to appear. We cannot exclude that at least some spores were not mature and infective yet, which would lower the infection dose in our experiment.

**Frequency of IgM + B cells and myeloid cells did not differ in exposed fish of the three different rearing backgrounds.** The lack of frequency differences for these immune cells between groups means that the exposure of the parental generation (F0) to the causative parasite did not elicit a measurable shift in the offspring’s immune cell counts. This can be different for other pathogens or other cell types. Further investigations on potential differences on molecular and gene levels are needed. CD8^+^ T cells showed differences between W animals and both other groups, but the results were only significant before Bonferroni correction. Similarly, the CD8^+^ T cells of the W animals were significantly different between the control and exposed groups before Bonferroni correction. Further scRNA-seq data collected at 25 dpe show similar differences between W animals and all other groups (Coxon et al., in prep).Regarding the host-parasite interaction at the interface to environmental conditions and their effect on immunity, Karvonen et al. [69] reported that salmonids with wild origin perform better during parasitic infections and have better survivals compared with fish originating from farm. Authors demonstrated that enriched rearing conditions can influence both survival and disease resistance in aquaculture fish. In our study, fish experienced different early-life rearing environments prior to being acclimatized at our facility, and their parents had either been previously exposed to the parasite (W and W:F groups) or remained unexposed (F group). We hypothesized that TGIP could be transmitted to the offspring, and/or that early environmental conditions would shape their immune responsiveness, as previously reported [60]. Since clear differences were observed only in the W group compared to all others, our findings suggest that early-life experience of the F1 generation may play a more significant role than parental exposure.

**A similar infection course was detected in all three fish groups.** At each sampling timepoint, we analyzed one fish per aquarium for determination of parasite presence by qPCR. These analyses indicated that the prevalence for *T. bryosalmonae* ranged from at least 14.3% to at least 85.7% per aquarium. This is comparable to other studies in brown trout with a mean infection prevalence of 60% at 15°C and 43% at 12°C [62]. In our study, temperature was continuously set at 16°C. In contrast, rainbow trout infected with the same procedure and similar parasite concentration reported a 100% infection prevalence at 15°C and 67–83% at 12°C [31,34]. Brown trout in another study showed 100% infection prevalence already at 10 dpe with the same parasite concentration as used before and similar to the concentration used in our study (42 x 10^6^ total copies) [64] and the same authors, in a consecutive study, reached between 70 and 100% prevalence in brown trout YOY [20]. These differences in infection prevalence within the same species and between species point out that intrinsic, e.g., differences in immune response, as well as extrinsic factors, e.g., infectivity of spores released by bryozoa, can play substantial roles in infection success and biological responses. The infection of previous parental generations with the parasite has not shown evidence of influence on the prevalence of infection in the next generation.

**Complex parasite life cycle can complicate the experimental design.** A limiting factor intrinsic to this disease to date is the fact that it includes two hosts, freshwater bryozoans and fish. The parasite itself cannot be cultivated in vitro and is dependent on the bryozoan host for production of infective spores. Although complicated, the infection is still possible, as was already proven in other publications [41,57] and in our own experiment.

In summary, this study provides new insights into frequencies of IgM^+^ B cells, myeloid cells, and CD8^+^ T cells in the kidneys of brown trout, in different rearing conditions and with different pathogen exposure of parental and offspring generations. Our findings underscore the importance of further investigating the effects of rearing environments and parental experience on pathogen resistance in F1 generations.

## Supporting information

S1 Fig Conjugate controls for flow cytometry. First row shows gating strategy: First, irregularities in sample acquisition are gated out with a “Time” gate. A second gate FSC-A/ FSC-H allows for gating out doublets. The third gate, where the P1 population is gated (lymphocytes and myeloid cells), is based on FSC-A/ SSC-A. The second row displays conjugate controls, showing unspecific staining of secondary antibodies. The third row shows the full staining (primary antibodies plus matching secondary antibodies). Example from the 4 days post exposure (dpe) sampling. kLC = kappa light chain.
(TIF)

S2 Fig Exemplary alternative gating of IgM + , CD8+ and myeloid+ cells. **A.** Exemplary gating of IgM+ cells from the lymphocyte gate. Backgating shows position of total IgM+ cells in FSC-A vs. SSC-A plot. **B.** Exemplary gating of CD8 + cells from the lymphocyte gate. Backgating shows position of total CD8 + cells in FSC-A vs. SSC-A plot. **C.** Exemplary gating of myeloid+ cells from the lymphocyte gate vs. the large-cell gate. Backgating shows position of total myeloid+ cells in FSC-A vs. SSC-A plot.
(TIF)(TIF)(TIF)

S3 Fig kappa light chain staining. Representative examples of the irregularities in the kLC positive staining. The shown “B cell gate” was based on IgM and kLC positive stainings, but was omitted due to irregular staining patterns.
(TIF)

S4 Fig Analysis of immune cells in brown trout kidney from the three fish rearing backgrounds (W, W:F, F) with flow cytometry. Represented are the percentages of cells of each population, either IgM^+^ B cells, myeloid, or CD8^+^ T cells within the P1 gate population at each sampling timepoint of the experiment. Frequencies were measured by flow cytometry from freshly isolated kidney immune cells of brown trout. Symbols show individual euthanized fish (n = 1–3 per control and exposed group and timepoint). Due to technical problems, on sampling day 54 only data for CD8^+^ T cells and no IgM^+^ B cells and myeloid cells are available.
(TIF)

S1 Table Comparison between control and exposed animals. T-test results for difference in frequency of IgM^+^ B cells, myeloid cells and CD8^+^ T cells between the control and exposed groups for Wild, Wild:Farm and Farm groups.
(DOCX)

S2 Table Group differences in immune cell frequencies. T-test results for difference in frequency of IgM^+^ B cells, myeloid cells and CD8^+^ T cells between the control and exposed animals for Wild, Wild:Farm and Farm groups, respectively.
(DOCX)
