# Supplementary figures and images for: Parental influence on brown trout offspring immune cell composition: An infection study with Tetracapsuloides bryosalmonae
Source: PLoS One. 2025 Sep 24;20(9):e0308779. doi: 10.1371/journal.pone.0308779 (PMC12459843; doi:10.1371/journal.pone.0308779)

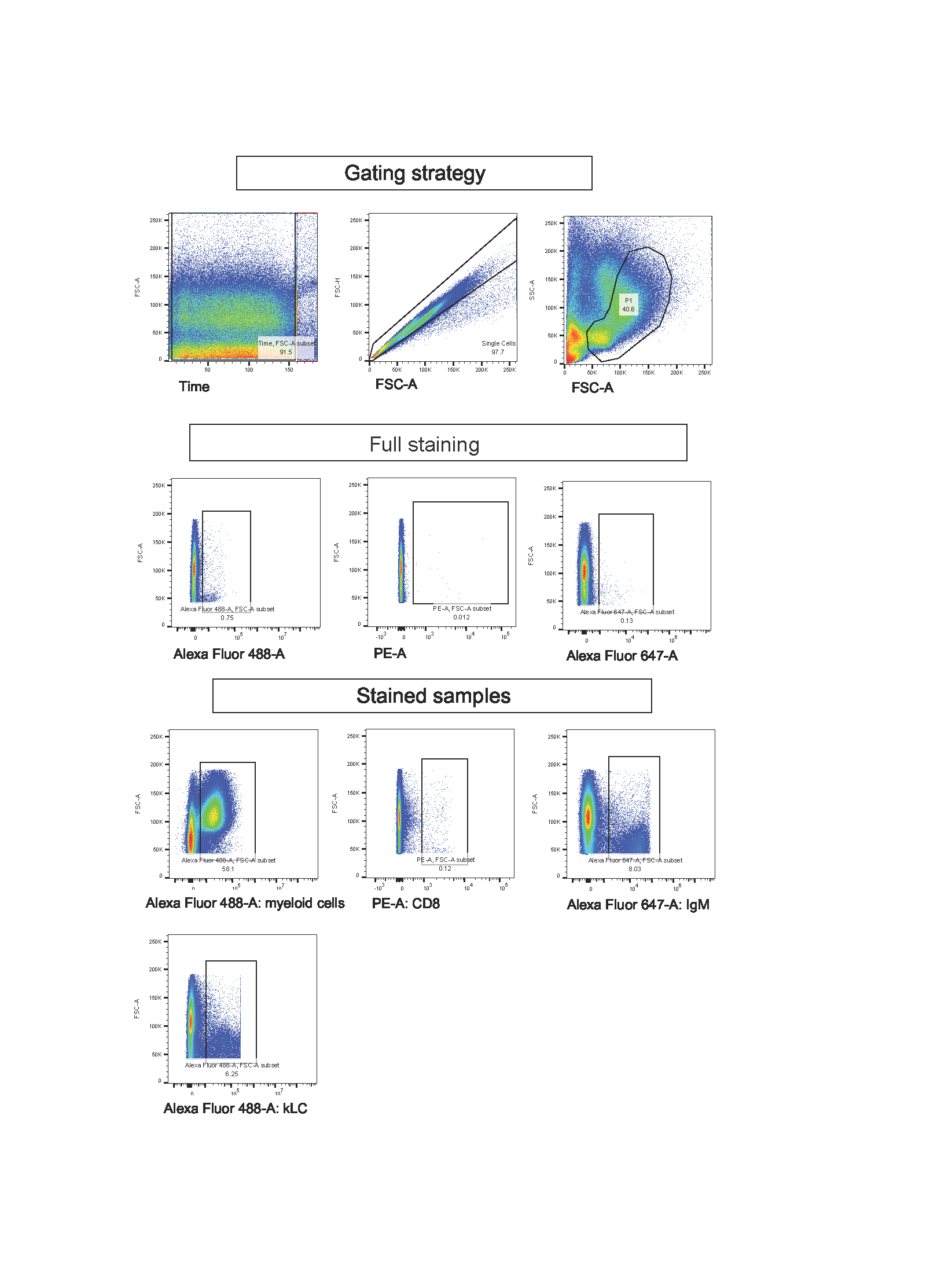

Supplement: S1 Fig — First row shows gating strategy: First, irregularities in sample acquisition are gated out with a “Time” gate. A second gate FSC-A/ FSC-H allows for gating out doublets. The third gate, where the P1 population is gated (lymphocytes and myeloid cells), is based on FSC-A/ SSC-A. The second row displays conjugate controls, showing unspecific staining of secondary antibodies. The third row shows the full staining (primary antibodies plus matching secondary antibodies). Example from the 4 days post exposure (dpe) sampling. kLC = kappa light chain. (TIF) [file pone.0308779.s001.tif]

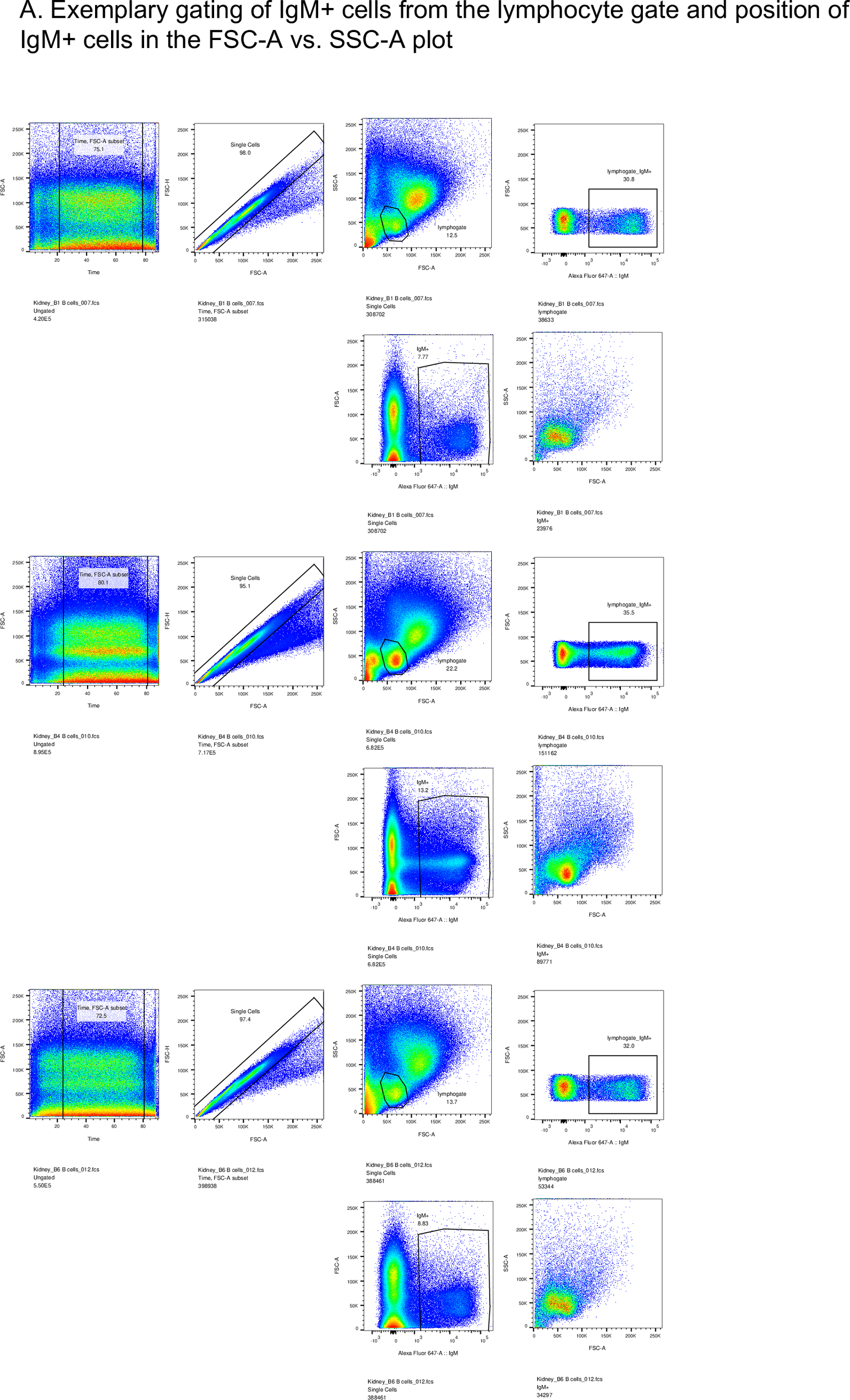

Supplement: S2 Fig — A. Exemplary gating of IgM+ cells from the lymphocyte gate. Backgating shows position of total IgM+ cells in FSC-A vs. SSC-A plot. B. Exemplary gating of CD8 + cells from the lymphocyte gate. Backgating shows position of total CD8 + cells in FSC-A vs. SSC-A plot. C. Exemplary gating of myeloid+ cells from the lymphocyte gate vs. the large-cell gate. Backgating shows position of total myeloid+ cells in FSC-A vs. SSC-A plot. (TIF)(TIF)(TIF) [file pone.0308779.s002.tif]

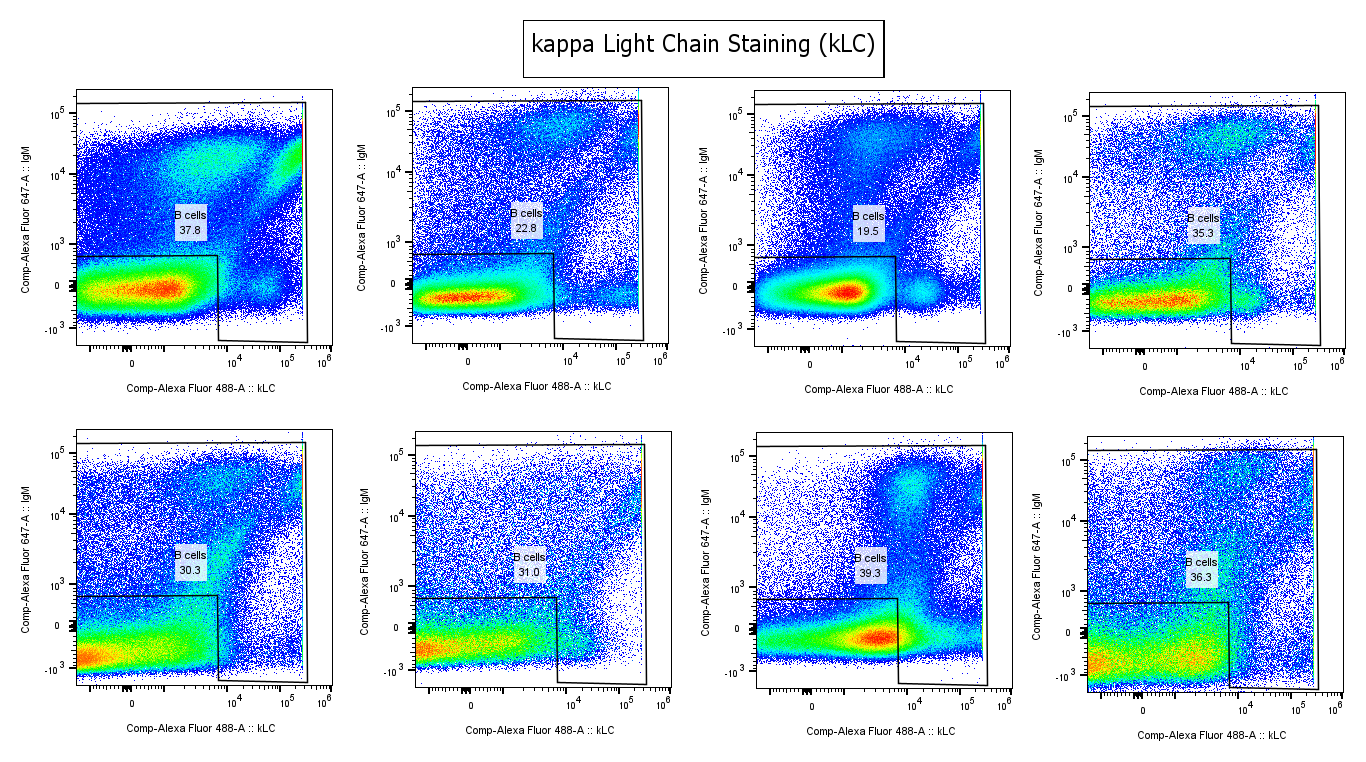

Supplement: S3 Fig — Representative examples of the irregularities in the kLC positive staining. The shown “B cell gate” was based on IgM and kLC positive stainings, but was omitted due to irregular staining patterns. (TIF) [file pone.0308779.s003.tif]

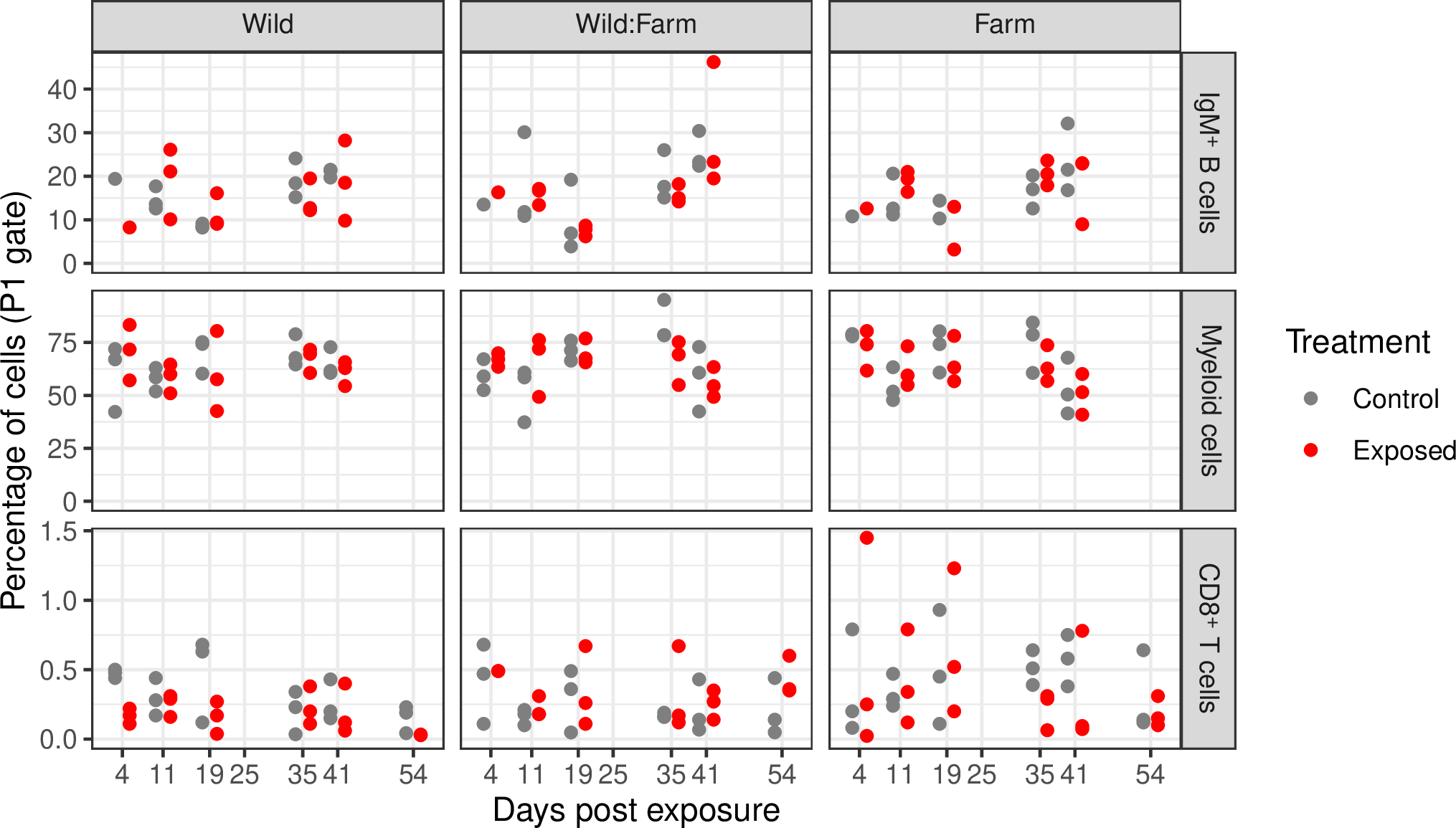

Supplement: S4 Fig — Represented are the percentages of cells of each population, either IgM+ B cells, myeloid, or CD8+ T cells within the P1 gate population at each sampling timepoint of the experiment. Frequencies were measured by flow cytometry from freshly isolated kidney immune cells of brown trout. Symbols show individual euthanized fish (n = 1–3 per control and exposed group and timepoint). Due to technical problems, on sampling day 54 only data for CD8+ T cells and no IgM+ B cells and myeloid cells are available. (TIF) [file pone.0308779.s004.tif]
