# Supplementary material for: Parental influence on brown trout offspring immune cell composition: An infection study with Tetracapsuloides bryosalmonae
Source: PLoS One. 2025 Sep 24;20(9):e0308779. doi: 10.1371/journal.pone.0308779 (PMC12459843; doi:10.1371/journal.pone.0308779)
Supplement: S1 Table — T-test results for difference in frequency of IgM+ B cells, myeloid cells and CD8+ T cells between the control and exposed groups for Wild, Wild:Farm and Farm groups. (DOCX) [file pone.0308779.s005.docx]

**Supplementary Table 1**: Comparison between control and exposed animals

| **Cell subtype** | **Group** | **T statistic** | **Degrees of freedom** | **p value** |
| --- | --- | --- | --- | --- |
| IgM+ B cells | W | -0.27 | 22.8 | .792 |
|  | W:F | -0.18 | 23.3 | .858 |
|  | F | 0.08 | 22.0 | .939 |
| Myeloid cells | W | -0.31 | 27.6 | .758 |
|  | W:F | -0.03 | 23.5 | .973 |
|  | F | -0.73 | 26.5 | .473 |
| CD8+ T cells | W | -2.44 | 28.5 | .021 |
|  | W:F | 1.44 | 32.8 | .159 |
|  | F | -0.30 | 28.5 | .766 |

T-test results for difference in frequency of IgM^+^ B cells, myeloid cells and CD8^+^ T cells between the control and exposed groups for Wild, Wild:Farm and Farm groups.
