# Supplementary material for: Parental influence on brown trout offspring immune cell composition: An infection study with Tetracapsuloides bryosalmonae
Source: PLoS One. 2025 Sep 24;20(9):e0308779. doi: 10.1371/journal.pone.0308779 (PMC12459843; doi:10.1371/journal.pone.0308779)
Supplement: S2 Table — T-test results for difference in frequency of IgM+ B cells, myeloid cells and CD8+ T cells between the control and exposed animals for Wild, Wild:Farm and Farm groups, respectively. (DOCX) [file pone.0308779.s006.docx]

**S2_Table**: Group differences in immune cell frequencies

| **Treatment** | **Cell subtype** | **Groups compared** | **T statistic** | **Degrees of freedom** | **p value** |
| --- | --- | --- | --- | --- | --- |
| Control | IgM+ B cells | W and W:F | -0.61 | 20.3 | .550 |

|  |  | W and F | -0.25 | 21.7 | .807 |
| --- | --- | --- | --- | --- | --- |
|  |  | W:F and F | 0.37 | 22.1 | .713 |
|  | Myeloid cells | W and W:F | -0.09 | 24.1 | .927 |
|  |  | W and F | -0.41 | 25.0 | .687 |
|  |  | W:F and F | -0.26 | 27.9 | .797 |
|  | CD8+ T cells | W and W:F | 1.03 | 34.0 | .310 |
|  |  | W and F | -1.56 | 31.4 | .128 |
|  |  | W:F and F | -2.45 | 30.9 | .020 |
| Exposed | IgM+ B cells | W and W:F | -0.50 | 21.0 | .624 |
|  |  | W and F | -0.54 | 23.0 | .594 |
|  |  | W:F and F | 0.08 | 20.5 | .940 |
|  | Myeloid cells | W and W:F | -0.39 | 27.3 | .700 |
|  |  | W and F | 0.10 | 28.0 | .924 |
|  |  | W:F and F | 0.49 | 27.2 | .628 |
|  | CD8+ T cells | W and W:F | -2.91 | 26.8 | .007 |
|  |  | W and F | -2.11 | 19.9 | .048 |
|  |  | W:F and F | -0.54 | 24.1 | .596 |

Legend S2_Table. T-test results for difference in frequency of IgM^+^ B cells, myeloid cells and CD8^+^ T cells between the control and exposed animals for Wild, Wild:Farm and Farm groups, respectively.
